# Evaluation of left ventricular dysfunction by three-dimensional speckle-tracking echocardiography and bioinformatics analysis of circulating exosomal miRNA in obese patients

**DOI:** 10.1186/s12872-023-03502-6

**Published:** 2023-09-11

**Authors:** Fuxin Wan, Xin Ma, Jiana Wang, Zhaohui An, Jiewen Xue, Qin Wang

**Affiliations:** 1 Clinical Medicine School, Ningxia Medical University, Yinchuan, Ningxia 750004 China; 2 Department of Cardiac Function Examination of Heart Centre, General Hospital of Ningxia Medical University, Yinchuan, Ningxia 750004 China

**Keywords:** Obesity, Left ventricular dysfunction, Echocardiography, Three-dimensional speckle-tracking echocardiography, Exosomal miRNA, Sequencing

## Abstract

**Background:**

Obesity is an independent risk factor for cardiovascular disease and affects the human population. This study aimed to evaluate left ventricular (LV) dysfunction in obese patients with three-dimensional speckle-tracking echocardiography (3D-STE) and investigate the possible related mechanisms at the exosomal miRNA level.

**Methods:**

In total, 43 participants (16 obese patients and 27 healthy volunteers) were enrolled. All subjects underwent full conventional echocardiography as well as 3D-STE. Characterization and high-throughput sequencing for the isolated circulating exosomes and the differentially expressed miRNAs (DEMs) were screened for target gene prediction and enrichment analysis.

**Results:**

Obese patients had significantly lower global longitudinal strain (GLS) (-20.80%±3.10% vs. -14.77%±2.05%, *P* < 0.001), global circumferential strain (GCS) (-31.63%±3.89% vs. -25.35%±5.66%, *P* = 0.001), global radial strain (GRS) (43.21%±4.89% vs. 33.38%±3.47%, *P* < 0.001), and indexed LV end-diastolic volume (LVEDV) [38.07mL/m^2^ (27.82mL/m^2^–9.57mL/m^2^) vs. 24.79mL/m^2^ (21.97mL/m^2^–30.73mL/m^2^), *P* = 0.002] than healthy controls. GLS (ρ = 0.610, *P* < 0.001), GCS (ρ = 0.424, *P* = 0.005), and GRS (ρ = -0.656, *P* < 0.001) indicated a moderate relationship with body mass index (BMI). In obese patients, 33 exosomal miRNAs were up-regulated and 26 exosomal miRNAs were down-regulated when compared to healthy controls (*P* < 0.05). These DEMs possibly contribute to obesity-associated LV dysfunction through the PI3K-Akt signaling pathway. Important miRNAs, including miR-101-3p, miR-140-3p, and miR-99a-5p, have clinical utility in predicting early obesity-related myocardial injury.

**Conclusions:**

The global strain obtained from 3D-STE can sensitively detect the decrease in LV myocardial function in obese patients. Key miRNAs and pathways provide a new theoretical basis and targets of action for studying obesity-induced LV dysfunction.

**Trial registration:**

In accordance with the World Health Organization (WHO) definition of a clinical trial, this study does not include human health-related interventions. This study was carried out at the General Hospital of Ningxia Medical University after obtaining institutional ethical approval (KYLL-2022-0556) and written informed consent from all participants.

## Background

Obesity is an independent risk factor for cardiovascular disease that can directly promote atherosclerosis, including cardiovascular disease morbidity and mortality [1]. Before the clinical manifestation of cardiovascular disease, obese individuals undergo structural and functional heart alterations due to obesity-induced chronic inflammation [2]. This chronic inflammation leads to oxidative stress, upregulated expression of inflammatory factors, and myocardial injury, which reduces cardiac systolic function [3, 4]. Conventional echocardiography inadequately detects subclinical changes in left ventricular (LV) function in obese patients, and reliance solely on LV ejection fraction (LVEF) proves inaccurate [5]. Three-dimensional speckle tracking echocardiography (3D-STE), an angle-independent imaging technique, assesses myocardial fiber displacement, global strain, and wall motion abnormalities, offering enhanced sensitivity to detect myocardial systolic function alterations [6, 7].

Understanding the signaling pathways underlying obesity-related cardiovascular disease is vital for early myocardial damage control. Exosomes, small extracellular vesicles, participate in intercellular communication and contain mass miRNAs (21–23 nucleotide non-coding RNA molecules). Exosomes can influence cardiovascular disease development and progression by releasing miRNAs in recipient cells to regulate target genes [8, 9]. The exosomal membrane shields miRNAs from degradation, making them stable and detectable in peripheral blood, potentially serving as biomarkers and therapeutic targets for cardiovascular diseases [10, 11].

This study aimed to assess LV dysfunction in obese patients using 3D-STE and to acquire circulating plasma exosomal miRNA expression profiles. Subsequent analysis was performed to obtain the theoretical basis and potential targets of obesity-induced LV dysfunction.

## Methods

### Participants

This prospective observational study was carried out at the General Hospital of Ningxia Medical University after obtaining institutional ethical approval (KYLL-2022-0556) and written informed consent from all participants. A total of 43 participants, aged between 18 and 50 years, were enrolled in the study, including 16 obese patients and 27 healthy volunteers. Obesity was diagnosed based on the criteria developed by the Working Group on Obesity in China (WGOC) and the expert consultation of the World Health Organization (WHO) [12], with the recruitment of patients confirmed to have a body mass index (BMI) greater than 28.0 kg/m^2^. Healthy volunteers were selected based on a stable BMI range of 18.5–23.9 kg/m^2^, normal physiological indexes, and systemic physical examinations conducted during the same period. Exclusion criteria for both groups were: (1) pregnancy or lactation; (2) secondary obesity caused by medication or other factors; (3) significant primary diseases, including hypertension, cardiovascular, cerebrovascular, hepatic, and renal diseases; (4) a history of gastrointestinal bariatric surgery; (5) undergoing fat loss therapy such as diet or exercise; and (6) addiction to smoking or alcohol consumption. Echocardiography was performed on all participants, and fasting venous whole blood was collected in the morning.

### Ultrasound measurements

All echocardiographic measurements were performed using a commercial scanner (EPIQ7C, Philips Ultrasound Co. Ltd., USA) in accordance with ASE/EACVI standards [13]. M-mode, two-dimensional echocardiography, and Doppler examinations were conducted using the S5-1 phased array probe (1–5 MHz). Interventricular septal end-diastolic thickness, LV posterior wall end-diastolic thickness, LV end-systolic diameters, and LV end-diastolic diameters were assessed by M-mode echocardiography, while LVEF was calculated. A flow Doppler sample volume was positioned at the tips of the mitral valve leaflets to measure early and late diastolic flow velocities. The early mitral annulus diastolic velocity was recorded by placing a tissue Doppler sample volume at the septal and lateral mitral annulus using a two-dimensional guided pulsed tissue Doppler cursor in the apical 4-chamber view.

3D-STE images were acquired from an apical position using an X5-1 matrix volume probe (1–5 MHz). Participants were instructed to breathe normally, and a full-volume dynamic image of the LV was obtained in the apical four-chamber cardiac view for three consecutive cardiac cycles. The data was analyzed using three-dimensional ultrasound image processing software (Tomtec, Philips Ultrasound Co. Ltd., Germany). The endocardial border was calibrated semi-automatically in LV analysis mode to obtain global longitudinal strain (GLS), global circumferential strain (GCS), and global radial strain (GRS) during LV systole, which provides information on myocardial deformation in different directions. Twist, torsion, LV end-diastolic volumes (LVEDV), and LV end-systolic volumes (LVESV) were simultaneously measured. Additionally, LVEDV and LVESV were reported both as absolute values and indexed to body surface area (BSA). To assess the agreement of global strain values, an additional experienced sonographer analyzed the same set of full-volume dynamic images, and the results were evaluated using Bland-Altman plots.

### Isolation and characterization of exosomes

Six exosome samples were extracted—three from healthy controls and three from obese patients. Venous blood was collected in the morning after an overnight fast and plasma was separated. Exosomes were isolated from plasma using an ultracentrifuge (CP100MX, Hitachi, Japan). Plasma was first centrifuged at 10,000 rpm for 45 min at 4 °C to remove larger vesicles. The supernatant was filtered through a 0.45 μm membrane to eliminate larger particles and then centrifuged at 100,000 rpm for 70 min at 4 °C. The resulting particles were resuspended in 10 mL of pre-chilled PBS and subjected to another centrifugation at 100,000 rpm for 70 min at 4 °C. The collected precipitate represented exosomes after removing the supernatant. Exosomes were characterized using transmission electron microscopy (TEM) (HT-7700, Hitachi, Japan) and nanoflow cytometry (NanoFCM) (N30E, Xiamen, China).

### Small RNA library construction and miRNA sequencing

Extraction of total RNA from exosomes by the Trizol method. Small RNA libraries were constructed and miRNA sequencing followed Illumina’s standard steps. TruSeq Small RNA Sample Prep Kits (Illumina, San Diego, CA, USA) were used for library preparation, and Illumina Hiseq2500 generated 1 × 50 bp single-end reads. Clean reads were obtained by processing raw reads using ACGT101-miR (LC Sciences, Houston, TX, USA). These clean reads were length-filtered to retain sequences of 18–26 nt and then filtered against mRNA, RFam, and Repbase databases to obtain valid data.

### Analysis of the DEMs

The miRNAs are clustered according to the similarity of sample miRNA expression profiles, and the clustering heat map of differentially expressed miRNAs (DEMs) is used to visualize the expression of miRNAs in different samples. The overall distribution of DEMs can be understood by drawing volcanic maps.

### Target genes prediction and enrichment

Target genes of DEMs were predicted by TargetScan (v5.0) and miRanda (v3.3a) software. TargetScan removes genes with context scores below the 50th percentile, while miRanda excludes those with Max Energy above -10. The final differential miRNA targets were chosen from the intersection of both software programs. To study the function of target mRNAs of DEMs, the Gene Ontology (GO, http://www.geneontology.org) and the Kyoto Encyclopedia of Genes and Genomes (KEGG, http://www.genome.jp/kegg) enrichment analyses [14–16] of target genes were performed using a hypergeometric validation approach.

### Statistical analysis

Statistical analysis was performed using SPSS 26.0 software. The Shapiro-Wilk test was used to test the normality of the measurement data. Normally distributed data were presented as the mean ± SD, and nonnormally distributed data were presented as the median (IQR). The student’s t-test was used for parametric variables, while the Mann-Whitney U and Chi-square tests were applied to estimate nonparametric variables for differences between the two groups. Correlations between continuous variables were tested using Spearman’s rho. The agreement was evaluated using Bland-Altman plots. All reported *P* values were two-sided, and statistical significance was defined as *P* < 0.05.

## Results

### Clinical characteristics

The clinical characteristics of all subjects are summarized in Table 1. The two groups were similar in terms of gender, age, systolic pressure, heart rate, and glucose. However, the obese patients had a higher BMI, diastolic pressure, cholesterol, triglycerides, and low-density lipoprotein and a lower high-density lipoprotein when compared to the healthy control.

**Table 1:** Clinical Characteristics for Healthy Control and Obese Patients

Characteristics	Healthy Control (n = 27)	Obese Patients (n = 16)	*P*
Gender (male)	17	12	0.342
Age (years)	33.44 ± 6.70	32.44 ± 8.03	0.661
BMI (kg/m^2^)	21.60 (20.10–22.40)	29.69 (29.02–32.51)	< 0.001
Systolic pressure (mmHg)	120.48 ± 11.55	125.75 ± 9.66	0.133
Diastolic pressure (mmHg)	73.11 ± 5.66	82.25 ± 4.97	< 0.001
Heart rate (beats/min)	67.70 ± 5.64	65.00 ± 8.21	0.208
GLU (mmol/L)	4.12 ± 0.64	4.46 ± 0.57	0.088
CHOL (mmol/L)	3.75 ± 0.75	4.28 ± 0.88	0.042
TG (mmol/L)	1.09 ± 0.43	1.53 ± 0.55	0.006
LDL (mmol/L)	2.16 ± 0.65	3.06 ± 0.64	< 0.001
HDL (mmol/L)	1.42 ± 0.40	0.96 ± 0.18	< 0.001

BMI body mass index, GLU Glucose, CHOL Cholesterol, TG Triglycerides, LDL Low-density lipoprotein, HDL Low-density lipoprotein

### Conventional echocardiographic parameters

As shown in Table 2, compared to the healthy control, the obese patients had the significantly higher interventricular septal end-diastolic thickness and LV posterior wall end-diastolic thickness. There were no significant differences between the groups for the other conventional echocardiographic parameters.

**Table 2:** Conventional Echocardiographic Parameters for Healthy Control and Obese Patients

Parameters	Healthy Control (n = 27)	Obese Patients (n = 16)	*P*
IVSD (mm)	7.62 ± 0.45	8.56 ± 0.65	< 0.001
LVPWD (mm)	7.66 ± 0.76	8.21 ± 0.54	0.015
LVEDD (mm)	47.17 ± 4.70	48.99 ± 3.67	0.192
LVESD (mm)	29.14 ± 3.71	31.48 ± 4.13	0.062
LVEF (%)	67.93 ± 5.06	64.88 ± 7.59	0.166
E (cm/s)	84.41 ± 15.86	87.68 ± 15.28	0.512
A (cm/s)	56.30 (47.10–64.50)	62.35 (56.90–84.08)	0.083
E/A	1.40 (1.20–1.70)	1.30 (1.20–1.58)	0.376
e’ (cm/s)	10.50 (9.20–14.80)	11.20 (9.10–12.45)	0.530
E/e’	7.30 (5.60–8.90)	8.15 (6.28–9.05)	0.274

IVSD interventricular septal end-diastolic thickness, LVPWD left ventricular posterior wall end-diastolic thickness, LVEDD left ventricular end-diastolic diameters, LVESD left ventricular end-systolic diameters, LVEF left ventricular ejection fraction, E early diastolic transmitral velocity, A late diastolic transmitral velocity, E/A early-to-late diastolic velocity ratio, e’ early mitral annulus diastolic velocity, E/e’ early diastolic transmitral velocity to early mitral annulus diastolic velocity ratio

### LV 3D-STE parameters

**Fig. 1:**
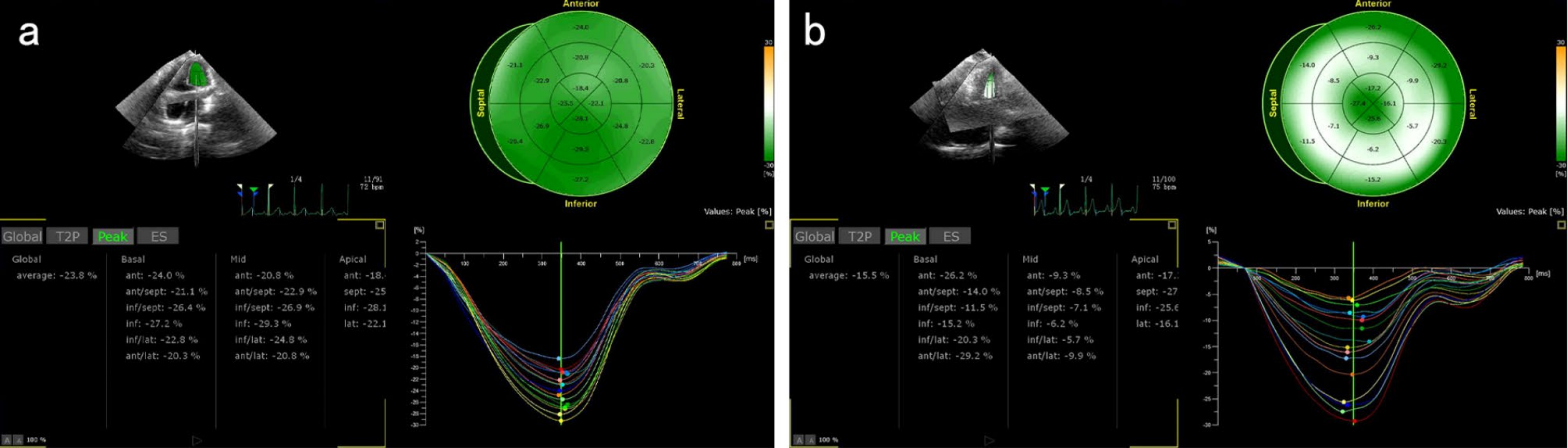
Global strain curves of healthy control (**a**) and obese patients (**b**)

3D-STE parameters are shown in Table 3. GLS, GCS, GRS, and indexed LVEDV were significantly lower in obese patients compared to the healthy control, whereas there were no significant differences between the groups for twist, torsion, LVEDV, LVESV, and indexed LVESV. In the healthy control, the strain curves were regular and the peak times were consistent; in the obese patients, the strain curves were disordered, with significantly lower peaks and inconsistent peak times (Fig. 1).

**Fig. 2:**
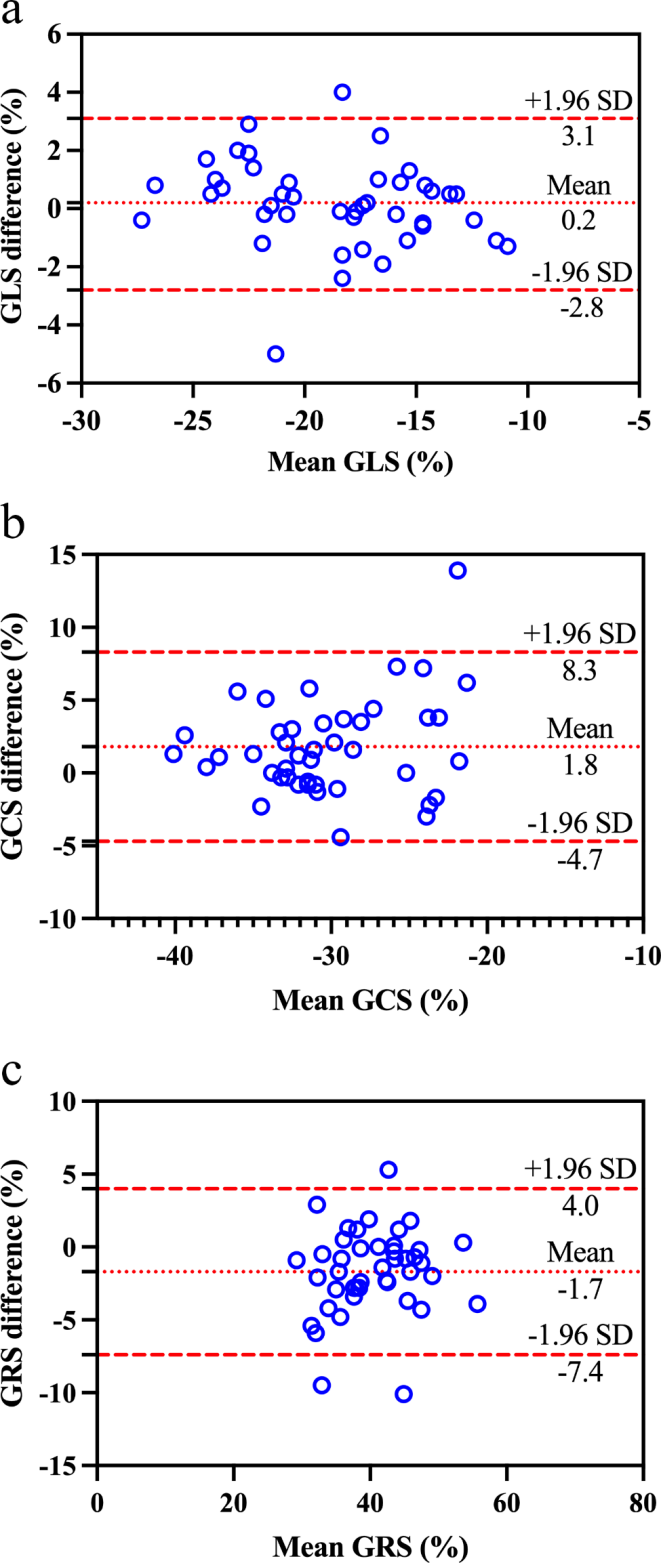
Bland-Altman plot of the global longitudinal strain (GLS) (**a**), global circumferential strain (GCS) (**b**), and global radial strain (GRS) (**c**)

Table 4 shows the correlation between global strain and clinical characteristics. GLS, GCS, and GRS indicated a moderate relationship with BMI (ρ = 0.610, *P* < 0.001; ρ = 0.424, *P* = 0.005; ρ = -0.656, *P* < 0.001, respectively). No significant correlations with gender, age, or height were found for GLS, GCS, and GRS. Bland-Altman plots showed narrow limits of agreement for global strain (Fig. 2). The middle-dotted line is the mean of the difference in measures. The upper and lower dashed lines are 1.96 standard deviations.

**Table 3:** LV 3D-STE Parameters for Healthy Control and Obese Patients

Parameters	Healthy Control (n = 27)	Obese Patients (n = 16)	*P*
GLS (%)	-20.80 ± 3.10	-14.77 ± 2.05	< 0.001
GCS (%)	-31.63 ± 3.89	-25.35 ± 5.66	0.001
GRS (%)	43.21 ± 4.89	33.38 ± 3.47	< 0.001
Twist (°)	14.32 ± 6.68	13.44 ± 6.01	0.666
Torsion (°)	1.78 ± 0.80	1.56 ± 0.69	0.360
LVEDV (mL)	68.12 ± 29.26	55.91 ± 16.81	0.090
LVESV (mL)	22.31 ± 11.64	21.38 ± 6.24	0.770
Indexed LVEDV (mL/m^2^)	38.07 (27.82–49.57)	24.79 (21.97–30.73)	0.002
Indexed LVESV (mL/m^2^)	12.17 (8.18–16.91)	9.68 (8.29–11.57)	0.108

GLS global longitudinal strain, GCS global circumferential strain, GRS global radial strain, LVEDV left ventricular end-diastolic volume, LVESV left ventricular end-systolic volume

**Table 4:** Correlation between Global Strain and Clinical Characteristics

Characteristics	GLS		GCS		GRS	
	ρ	*P*	ρ	*P*	ρ	*P*
Gender	0.014	0.930	0.147	0.345	-0.051	0.745
Age	-0.213	0.170	-0.137	0.380	-0.149	0.339
Height	-0.036	0.818	0.046	0.771	-0.060	0.701
BMI	0.610	< 0.001	0.424	0.005	-0.656	< 0.001

### Characterization of isolated exosomes

**Fig. 3:**
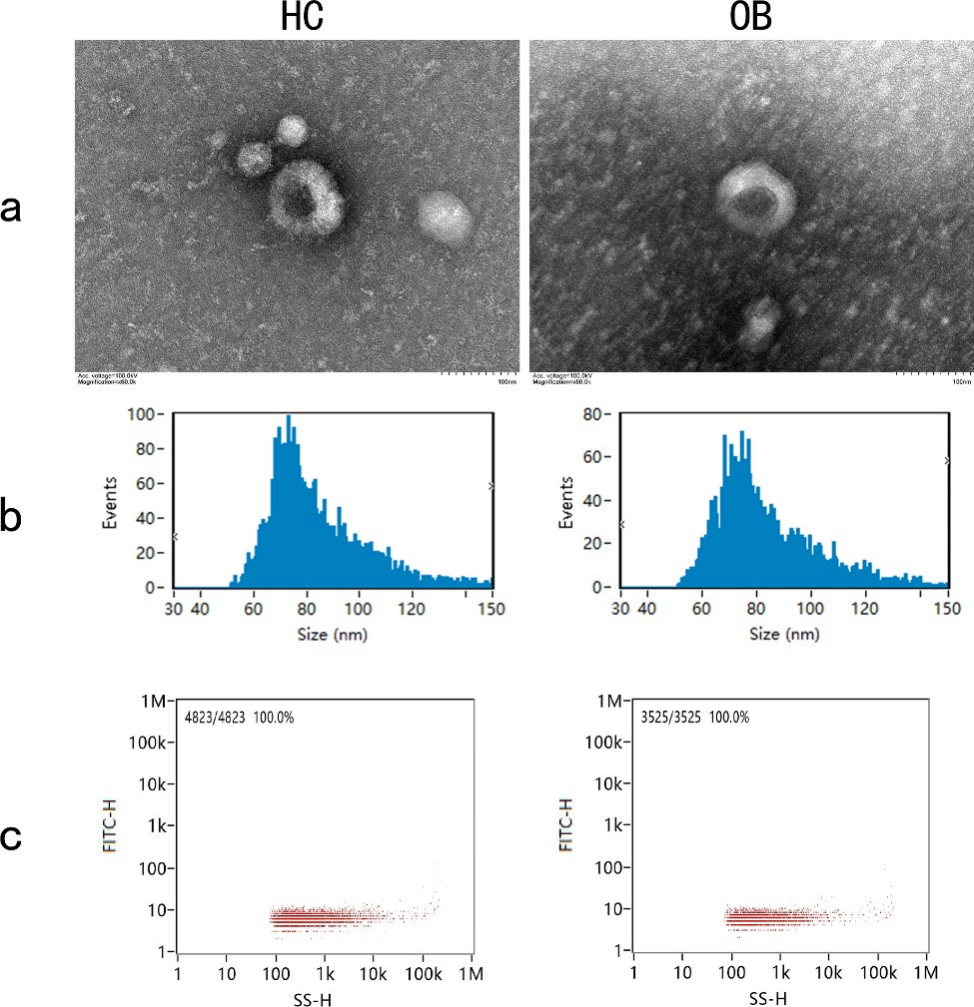
Characterization of plasma exosomes from healthy control (HC) and obese patients (OB). Morphology of exosomes was observed by transmission electron microscopy (TEM) (**a**). Size distribution was analyzed by NanoFCM (**b**). Particle concentration was analyzed by NanoFCM (**c**)

The plasma samples from the two groups were collected and used for exosome isolation. As shown in Fig. 3, the typical morphological structure of exosomes can be observed by TEM. NanoFCM revealed that the particle size distribution of the two groups was primarily between 30 and 150 nm, with concentrations of 2.46 × 10^9^ particles/ml and 3.57 × 10^9^ particles/ml.

### Analysis of the DEMs

**Fig. 4:**
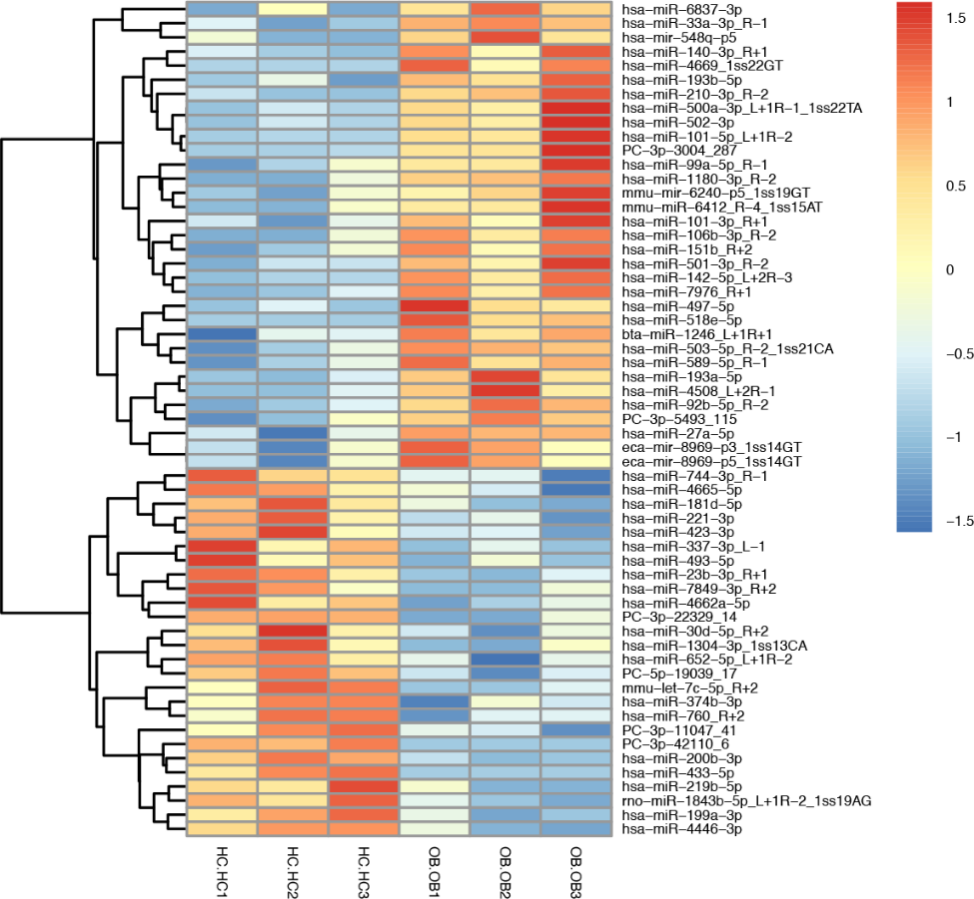
Heatmap of DEMs. The miRNA expression levels are expressed as log10-transformed normalized values. Different colors represent different levels of miRNA expression. The colors range from blue through white to red, representing low to high levels of expression. Red indicates high expression, and dark blue indicates low expression

**Fig. 5:**
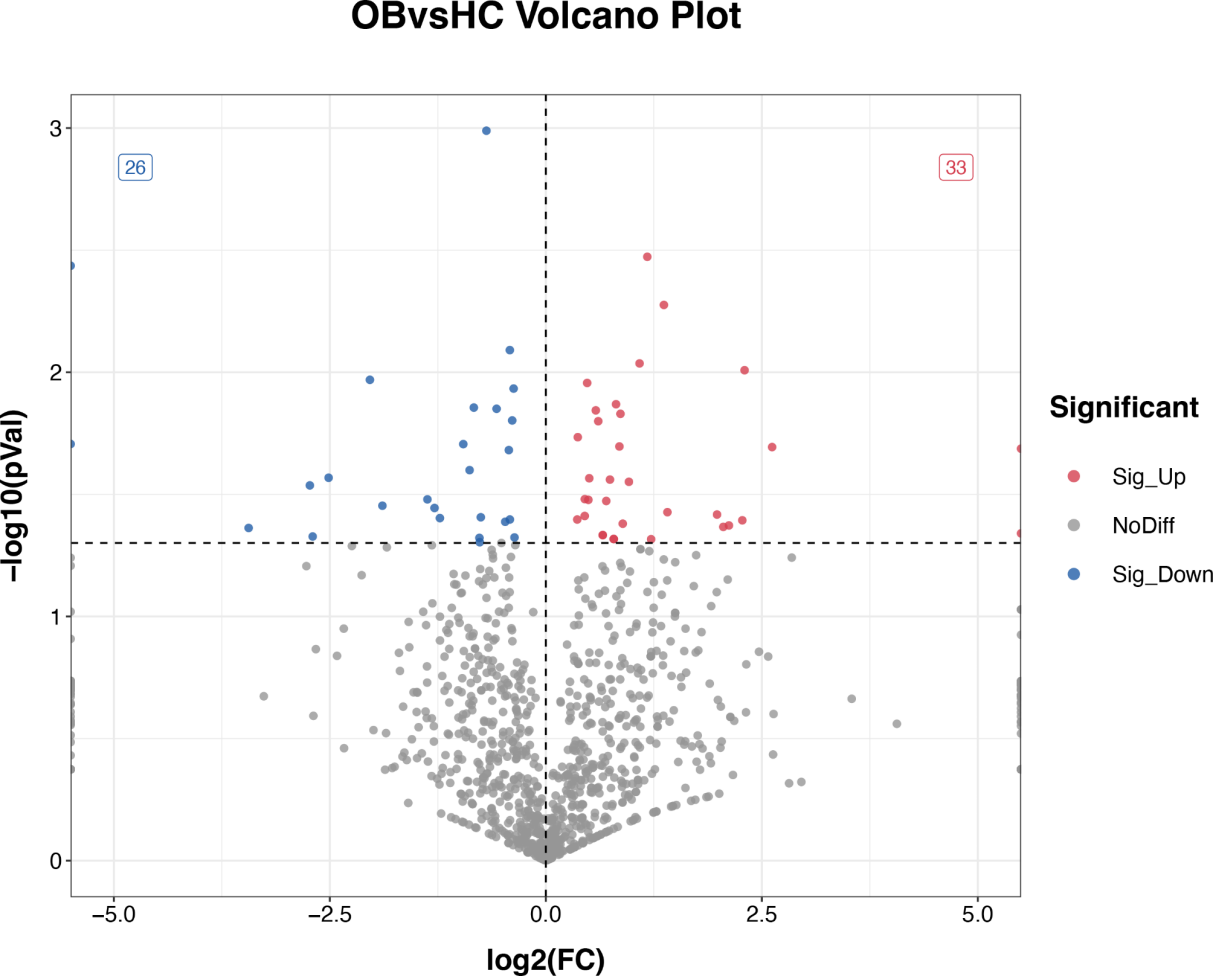
Volcano plot of DEMs. log2(FC) represents miRNA differential expression fold change in different samples; -log10(*p*-value) represents the statistical significance of the difference in miRNA expression; red represents up-regulated significantly DEMs, blue represents down-regulated significantly DEMs, and grey dots represent non-significant DEMs

A total of 1611 DEMs were detected, and six new miRNAs were predicted, namely PC-3p-5493_115, PC-3p-3004_287, PC-3p-11047_41, PC-5p-19039_17, PC-3p-22329_14, and PC-3p-42110_6. Differential miRNA clustering analysis straightforwardly presented the specific expression profiles of the miRNAs in the two groups (Fig. 4), and the advanced volcano map presented the significant DEMs (Fig. 5). The data showed that, compared with the healthy controls, there were 33 miRNAs up-regulated and 26 miRNAs down-regulated significantly in the obese patients. The expression levels and the up- or down-regulation relationships of the top 20 DEMs are presented in Table 5. Target gene prediction and GO and KEGG enrichment analyses of these significant DEMs were subsequently performed.

**Table 5:** Top 20 DEMs and their expression levels

miRNA	Fold change	Log2 FC	*p*-value	Exp level	Up/down
hsa-miR-101-3p	1.85	0.89	1.43E-02	high	up
hsa-miR-106b-3p	1.49	0.58	1.84E-02	high	up
hsa-miR-140-3p	1.37	0.45	3.31E-02	high	up
hsa-miR-142-5p	1.29	0.37	4.01E-02	high	up
hsa-miR-99a-5p	1.29	0.36	4.17E-02	high	up
hsa-miR-193b-5p	4.93	2.30	3.37E-03	middle	up
mmu-miR-6412	4.34	2.12	5.30E-03	middle	up
hsa-miR-101-5p	4.15	2.05	9.20E-03	middle	up
PC-3p-5493_115	3.95	1.98	9.81E-03	middle	up
mmu-mir-6240-p5	2.65	1.41	1.11E-02	middle	up
hsa-miR-30d-5p	0.78	-0.36	1.17E-02	high	down
hsa-miR-199a-3p	0.77	-0.37	1.58E-02	high	down
hsa-miR-23b-3p	0.76	-0.39	2.08E-02	high	down
hsa-miR-221-3p	0.74	-0.43	4.10E-02	high	down
hsa-miR-423-3p	0.72	-0.47	4.75E-02	high	down
rno-miR-1843b-5p	0.75	-0.42	1.03E-03	middle	down
hsa-miR-181d-5p	0.67	-0.57	8.12E-03	middle	down
hsa-miR-200b-3p	0.62	-0.69	1.07E-02	middle	down
hsa-miR-4665-5p	0.59	-0.75	1.40E-02	middle	down
hsa-miR-374b-3p	0.59	-0.77	1.41E-02	middle	down

### Enrichment analysis of target genes

**Fig. 6:**
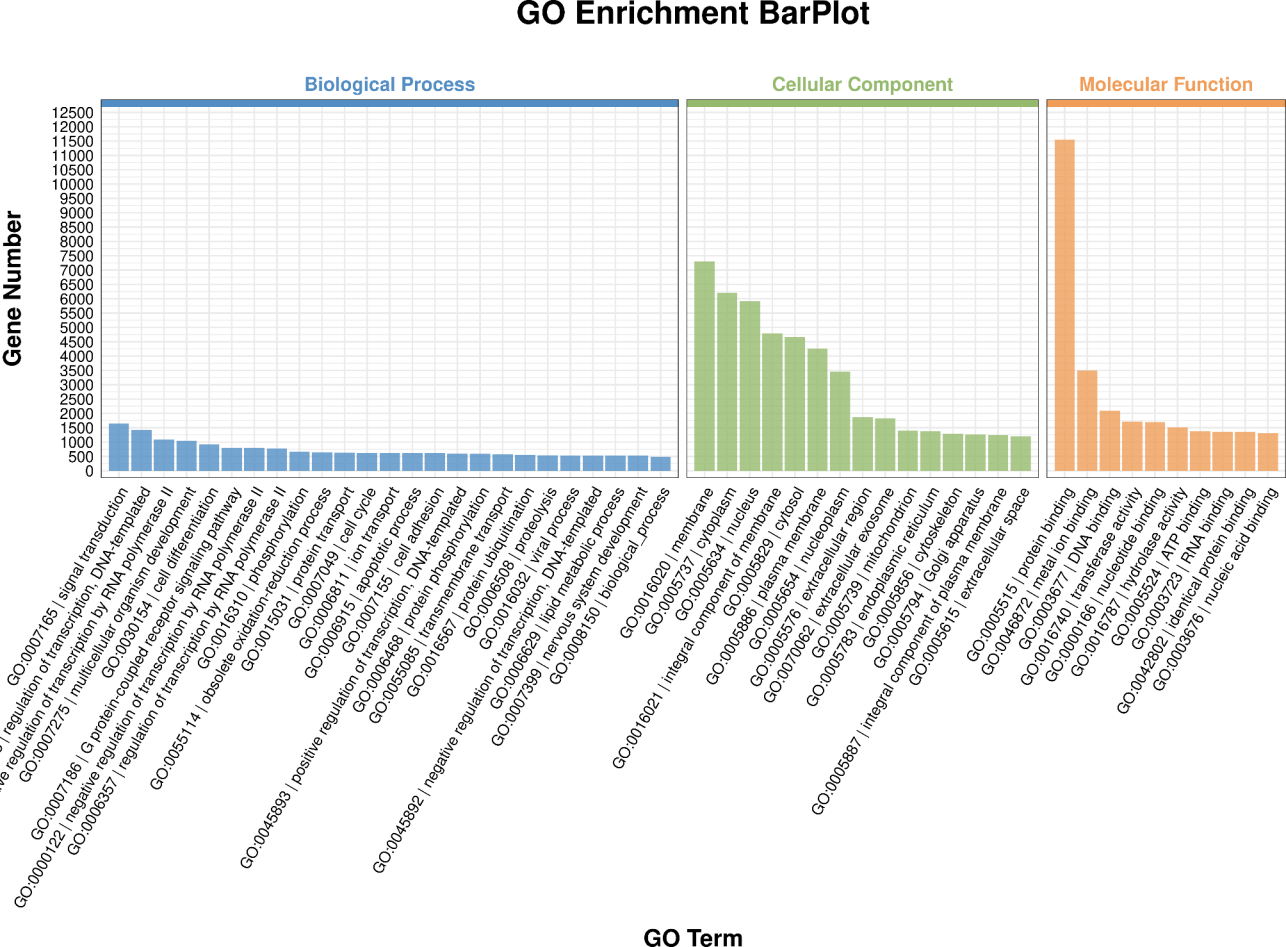
GO enrichment BarPlot. The horizontal coordinate is the classification of the GO term, and the vertical coordinate is the number of target genes

**Fig. 7:**
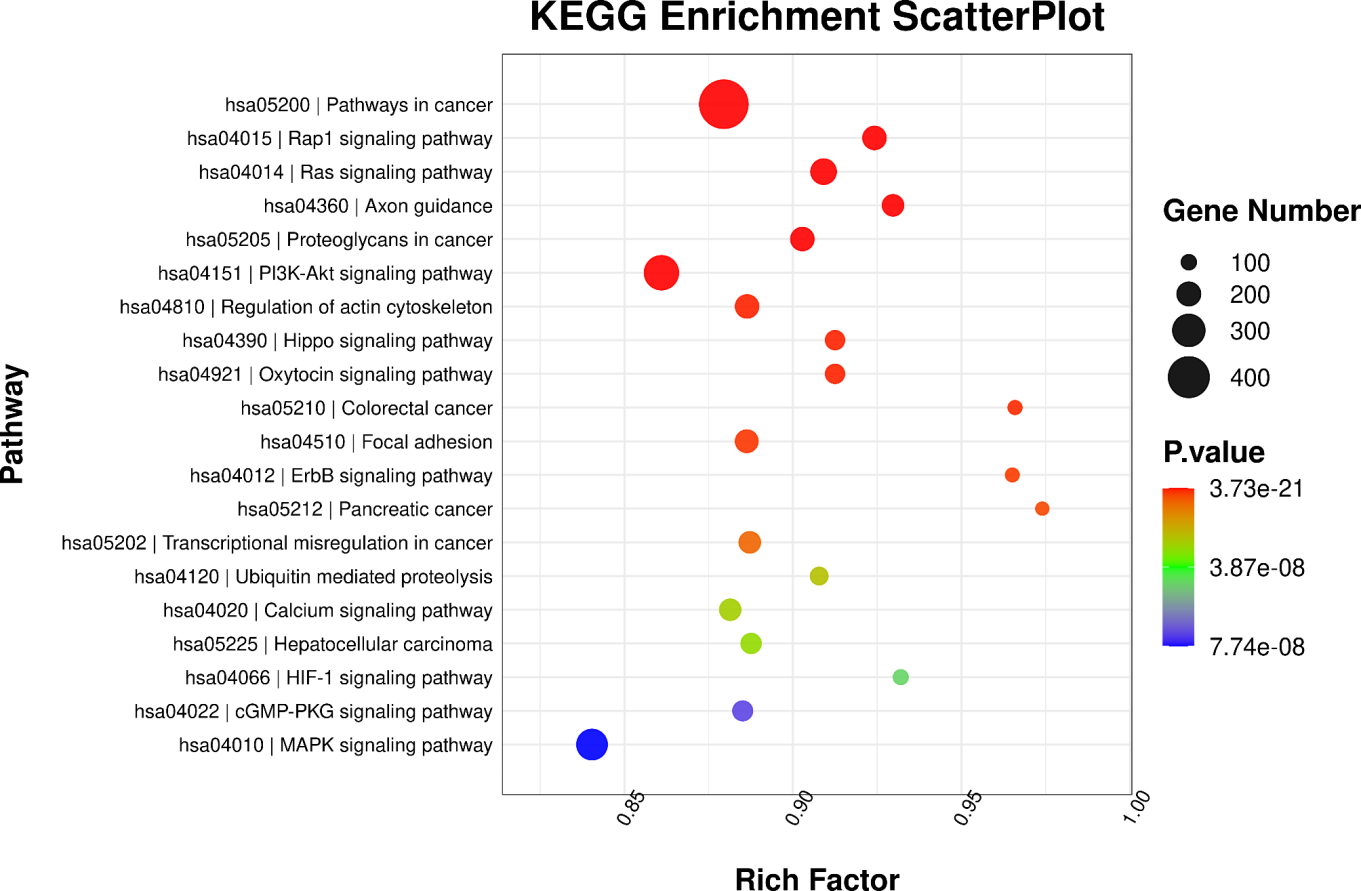
KEGG enrichment ScatterPlot. The Rich factor is the ratio of the number of target genes in the KEGG to the total number of genes in the KEGG; the higher the Rich factor, the greater the degree of KEGG enrichment. A phrase with a lower *p*-value has a higher degree of enrichment. The width of the point indicates the number of genes that have been enriched with the item

In total, 17,170 target genes were predicted using the TargetScan (v5.0) and miRanda (v3.3a) software. GO annotation and KEGG pathway analyses were performed to understand the biological function and key pathways of the target genes. Figure 6 depicts the findings of the GO enrichment analysis. The biological process (BP) enrichment analysis revealed that 1652, 1427, 1098, and 1044 target genes were involved in signal transduction, transcription regulation of the DNA template, positive transcription regulation by RNA polymerase II, and multicellular organism development, respectively. According to the cellular component (CC) enrichment analysis, 7297, 6203, and 5920 of the target genes were engaged in the composition of the membrane, cytoplasm, and nucleus, respectively. In addition, the molecular functional (MF) analysis revealed that the target genes were primarily involved in protein binding (11,549), metal ion binding (3496), and DNA binding (2094), respectively. Moreover, the KEGG enrichment analysis (Fig. 7) showed that these target genes were predominantly enriched in the PI3K-Akt signaling pathway, pathways in cancer, the Ras signaling pathway, the Rap1 signaling pathway, and axon guidance. This discovery is critical for our further screening of the target genes involved in an obesity-induced myocardial injury at the exosomal miRNA level.

## Discussion

In this study, we conducted a comprehensive examination of LV dysfunction in obese patients utilizing 3D-STE. Concurrently, we analyzed circulating exosomal miRNA expression profiles using advanced bioinformatics techniques. Our findings provide a unique insight into the cardiovascular changes linked to obesity. In this discussion, we will elucidate the interrelationships between these facets and their potential mechanisms, providing insights into the intricate relationship between obesity and LV dysfunction.

Compared with the healthy control, the interventricular septal end-diastolic thickness and LV posterior wall end-diastolic thickness were significantly higher in obese patients in the present study. Although these parameters remained within the normal range according to international diagnostic guidelines, they indicated relative ventricular wall hypertrophy resulting from abnormal fat accumulation in obese patients. Additionally, widely used echocardiographic indices for assessing LV systolic and diastolic function did not reveal significant distinctions between the two groups. These findings imply that these parameters may not effectively detect subclinical impairments in LV myocardial function because of their insufficient sensitivity [17].

Myocardial fibers are anatomically arranged in a spiral pattern, and they can be categorized into longitudinal, circumferential, and oblique orientations, extending from the endocardium to the epicardium [18]. These orientations correspond to GLS, GCS, and GRS, respectively. It is important to note that positive and negative strain values solely represent the direction of myocardial fiber motion.

Consistent with the findings of Doğduş M et al. [19], our study showed that GLS, GCS, and GRS were significantly reduced in obese patients compared to healthy controls. We also found a significant reduction in the indexed LVEDV in obese patients, which was not mentioned in the study of Doğduş M et al. [19]. These results suggest that LV dysfunction already exists in obese patients and that their hearts have undergone ventricular remodeling to accommodate the increased workload due to excess weight. Notably, twist, torsion, LVEDV, LVESV, and indexed LVESV did not exhibit statistically significant differences between the two groups. We speculate that this lack of difference may be attributed to the slow progress of myocardial damage and contractile function decline in response to obesity. During this process, the neurohumoral system may regulate the body, prompting compensatory contractions in the undamaged myocardium to maintain normal LV torsional movement and ejection fraction [20]. Additionally, we observed correlations between GLS, GCS, and GRS and BMI in this study (ρ = 0.610, *P* < 0.001; ρ = 0.424, *P* = 0.005; ρ = -0.656, *P* < 0.001, respectively). With increasing BMI, there was a corresponding decrease in the LV global strain index, indicating declining myocardial function.

In this study, we generated small RNA libraries for both two groups and performed miRNA-seq to sequence circulating exosomal miRNAs. We evaluated the sequencing data quality using the Q30 metric, and the results indicated that all samples achieved a Q30 score exceeding 95%, confirming the acquisition of high-quality small RNA libraries and sequencing data. Consequently, we observed significant differences in plasma exosomal miRNA expression levels between the two groups. In total, we identified 59 DEMs through a comparative analysis, consisting of 33 up-regulated and 26 down-regulated miRNAs in obese patients. Among these identified miRNAs, hsa-miR-101-3p, hsa-miR-106b-3p, hsa-miR-140-3p, hsa-miR-142-5p, and hsa-miR-99a-5p exhibited up-regulation and relatively high expression levels, rendering them promising candidates as markers and targets for further analysis.

Previous research has underscored the importance of specific miRNAs in different cardiac conditions. Xin et al. [21] found a link between increased miR-101-3p levels in the serum of sepsis-induced cardiomyopathy patients and pro-inflammatory cytokines such as IL-1β, IL-6, and TNF-α. Inhibiting miR-101-3p was shown to reduce inflammation and apoptosis by suppressing the MAPK p38 and NF-kB pathways, implying its role in myocardial inflammatory responses. Another study by Wei DZ et al. [22] demonstrated that upregulation of miR-140-3p expression promoted ventricular remodeling after acute myocardial infarction. Conversely, inhibiting the expression of miR-140-3p was found to alleviate the decrease in myocardial contractility induced by afterload [23]. Additionally, circulating miR-140-3p was a reliable predictor of cardiovascular death in patients with acute coronary syndrome [24]. Han Z et al. [25] found that miR-99a-5p overexpression inhibited aortic smooth muscle cell proliferation, migration, and invasion, which are linked to atherosclerotic disease development. By targeting the homeobox A1 gene, miR-99a-5p acted as an atherosclerosis inhibitor, reducing lesion formation. Recent research showed selective miR-99a-5p overexpression significantly decreased atherosclerotic lesions and lowered NLRP3 inflammatory protein expression [26], leading to reduced inflammasome complex and inflammatory cytokine levels. Overall, these significant miRNAs, including miR-101-3p, miR-140-3p, and miR-99a-5p, hold clinical utility for the early prediction of obesity-related myocardial injury.

The GO enrichment analysis revealed that most target gene products are involved in membrane, cytoplasm, and nucleus composition. Their molecular functions are primarily associated with protein binding, metal ion binding, and DNA binding. Additionally, these target genes participate in various biological processes, including signal transduction, transcriptional regulation of DNA templates, positive regulation of RNA polymerase II-mediated transcription, and multicellular organism development.

The KEGG enrichment analysis demonstrated that these target genes were predominantly enriched in the PI3K-Akt signaling pathway, pathways in cancer, the Ras signaling pathway, the Rap1 signaling pathway, and axon guidance. Of these pathways, the PI3K-Akt signaling pathway is pivotal in regulating cardiomyocyte growth and survival, and it plays a crucial role in cardiomyocyte ischemia and subsequent LV remodeling [27]. Numerous studies have highlighted the impact of the PI3K-Akt signaling pathway on myocardial fibrosis by modulating cell survival, apoptosis, growth, cardiac contractility, and gene transcription [28]. Consequently, it is reasonable to speculate that DEMs may govern processes such as obesity-related ventricular remodeling, neovascularization, and myocardial fiber formation through the PI3K-Akt signaling pathway, ultimately contributing to LV dysfunction.

### Limitations

This study possesses several potential limitations that warrant consideration. Firstly, the sample size utilized in our study was relatively small. This limitation may impact the generalizability of our findings to a larger population, reduce our statistical power, and hinder the detection of subtle but clinically existing differences. Secondly, it is crucial to acknowledge that the current experimental studies of miRNAs encounter challenges arising from the lack of standardized inter- and intra-laboratory processes. The absence of uniformity in experimental protocols hampers the establishment of miRNAs as reliable biomarkers and viable targets for therapeutic intervention. Lastly, key miRNAs and pathways need animal models or cell cultures for further validation. We will refine the relevant studies in subsequent experiments to further validate the present findings.

## Conclusions

In conclusion, our study highlights the intricate interplay between obesity, LV dysfunction, and plasma exosomal miRNA expression profiles. We have demonstrated that global strain measured by 3D-STE serves as a sensitive indicator of subclinical myocardial dysfunction in obese individuals, with a corresponding decline as BMI increases. Furthermore, significant differences in exosomal miRNA expression between obese patients and healthy controls, and DEMs possibly contribute to obesity-associated LV dysfunction through the PI3K-Akt signaling pathway. Important miRNAs, including miR-101-3p, miR-140-3p, and miR-99a-5p, have clinical utility in predicting early obesity-related myocardial injury. These findings not only establish a crucial theoretical basis but also identify potential therapeutic targets for further exploration in the context of obesity-related myocardial injury. Our study underscores the importance of continued research to unravel the intricate relationship between obesity and LV dysfunction, ultimately paving the way for more effective interventions and treatments in this population.

## Data Availability

The datasets generated and analyzed during the current study are available in the Sequence Read Archive (SRA) repository, direct link: https://dataview.ncbi.nlm.nih.gov/object/PRJNA989166?reviewer=qtqk40f6ngeufcfu05j6lesrp0, and accession ID: PRJNA989166. Additional data is available from the corresponding author upon reasonable request.

## Abbreviations

LV: Left ventricular
3D-STE: Three-dimensional speckle-tracking echocardiography
LVEF: Left ventricular ejection fraction
BMI: Body mass index
GLS: Global longitudinal strain
GCS: Global circumferential strain
GRS: Global radial strain
LVEDV: Left ventricular end-diastolic volumes
LVESV: Left ventricular end-systolic volumes
DEMs: Differentially expressed miRNAs
GO: Gene Ontology
KEGG: Kyoto Encyclopedia of Genes and Genomes
